# Adaptive Evolution Is Substantially Impeded by Hill–Robertson Interference in *Drosophila*

**DOI:** 10.1093/molbev/msv236

**Published:** 2015-10-22

**Authors:** David Castellano, Marta Coronado-Zamora, Jose L. Campos, Antonio Barbadilla, Adam Eyre-Walker

**Affiliations:** ^1^Genomics, Bioinformatics and Evolution Group, Institut de Biotecnologia i de Biomedicina (IBB) and Department de Genètica i Microbiologia, Campus Universitat Autònoma de Barcelona, Cerdanyola del Vallès, Spain; ^2^Institute of Evolutionary Biology, School of Biological Sciences, University of Edinburgh, Edinburgh, United Kingdom; ^3^Centre for the Study of Evolution, School of Life Sciences, University of Sussex, Brighton, United Kingdom

**Keywords:** Hill–Robertson, adaptation, recombination, mutation, *Drosophila*, gene density

## Abstract

Hill–Robertson interference (HRi) is expected to reduce the efficiency of natural selection when two or more linked selected sites do not segregate freely, but no attempt has been done so far to quantify the overall impact of HRi on the rate of adaptive evolution for any given genome. In this work, we estimate how much HRi impedes the rate of adaptive evolution in the coding genome of *Drosophila melanogaster.* We compiled a data set of 6,141 autosomal protein-coding genes from *Drosophila*, from which polymorphism levels in *D. melanogaster* and divergence out to *D. yakuba* were estimated. The rate of adaptive evolution was calculated using a derivative of the McDonald–Kreitman test that controls for slightly deleterious mutations. We find that the rate of adaptive amino acid substitution at a given position of the genome is positively correlated to both the rate of recombination and the mutation rate, and negatively correlated to the gene density of the region. These correlations are robust to controlling for each other, for synonymous codon bias and for gene functions related to immune response and testes. We show that HRi diminishes the rate of adaptive evolution by approximately 27%. Interestingly, genes with low mutation rates embedded in gene poor regions lose approximately 17% of their adaptive substitutions whereas genes with high mutation rates embedded in gene rich regions lose approximately 60%. We conclude that HRi hampers the rate of adaptive evolution in *Drosophila* and that the variation in recombination, mutation, and gene density along the genome affects the HRi effect.

## Introduction

It has been shown that there are substantial levels of adaptive protein evolution in many species; for example, in species of *Drosophila*, rodents, bacteria, and some plants, and it has been estimated that greater than 25% of all amino acid substitutions are consequence of positive adaptive evolution (Bustamante et al. 2002; Smith and Eyre-Walker 2002; Bierne and Eyre-Walker 2003; Sawyer et al. 2003; Charlesworth and Eyre-Walker 2006; Haddrill et al. 2010; Ingvarsson 2010; Slotte et al. 2010; Strasburg et al. 2011). In contrast, there are some species, such as humans and many other plants for which rates of adaptive evolution appear to be very low (Chimpanzee Sequencing and Analysis Consortium 2005; Zhang et al. 2005; Boyko et al. 2008; Eyre-Walker and Keightley 2009; Gossmann et al. 2010). The reason for this variation between species is not fully understood, although effective population size (*Ne*) appears to be important (Gossmann et al. 2012).

The rate of adaptive evolution also appears to vary between genes within a genome. This is expected for several reasons. First, some genes are expected to undergo more adaptive evolution because of their functions; in particular those genes that interact with the environment or which are caught up in arms races are expected to have high rates of adaptive evolution, whereas those genes with highly conserved functions are expected to adapt slowly. Second, genes with high mutation rates are predicted to adapt faster than those with low mutation rates. This is expected whether most adaptation comes from newly arising mutations or from standing genetic variation. This is obvious if adaptation is mutation limited; if an organism is waiting for advantageous mutations to arise, and adaptation can potentially occur in more than one gene, then adaptation is mostly likely to occur in the gene with the highest mutation rate. However, we also expect adaptation to be greater even if advantageous mutations are selected from standing genetic variation, because genes with the highest mutation rates will contribute most to diversity. Third, we expect the rate of adaptive evolution to depend upon the rate of recombination; genes with low rates of recombination will suffer from Hill–Robertson interference (HRi) (Hill and Robertson 1966; Felsenstein 1974) in which selected mutations interfere with each other: a newly arising advantageous mutation may find itself in linkage disequilibrium with deleterious mutations, which will reduce its probability of fixation if it cannot recombine away from them, or in competition for fixation with another advantageous mutation at a linked locus on another chromosome in the population. Fourth, we expect an interaction between the rate of recombination and the rate of mutation; HRi should be more prevalent in genes with high mutation rates and low rates of recombination. Fifth, following the same logic, genes embedded in gene rich regions should also show stronger HRi than genes located in gene poor regions and so HRi should be pervasive in genes with high mutation rates, high gene density, and low recombination.

Several studies have shown that gene function is important in determining the rate of adaptive evolution: Obbard et al. (2009) have shown that immune system genes have higher rates of adaptive evolution than other genes in *Drosophila,* and Haerty et al. (2007) and Pröschel et al. (2006) have shown that male-biased genes, like testes specific genes, have higher rates of adaptive evolution. It has also been shown that in humans many of the genes that present a signature of positive selection tend to be involved in sensory perception, immune defenses, tumor suppression, apoptosis, and spermatogenesis (Clark et al. 2003; Chimpanzee Sequencing and Analysis Consortium 2005; Nielsen et al. 2005). The role of recombination has also been studied; it has been shown in *Drosophila* that the rates of adaptation in different regions of the genome vary greatly by differences in the frequency of recombination (Presgraves 2005; Betancourt et al. 2009; Arguello et al. 2010; Mackay et al. 2012; Campos et al. 2014). Surprisingly, the role of the mutation rate and gene density in the rate of protein adaptive evolution has not been considered before.

Our analysis has shown how the rate of recombination, the mutation rate and gene density affect the rate of adaptation within the *Drosophila melanogaster* genome. We find that the rate of adaptive amino acid substitution is positively correlated to both recombination rate and an estimate of the mutation rate, whereas it is negatively correlated to the gene density. We also find that this correlation is robust to controlling for each other, synonymous codon bias and gene functions related to immune response and testes. Finally, we estimate that on average at least approximately 27% of all advantageous substitutions have been lost because of HRi and that this quantity depends on gene’s mutation rate and the gene density where the gene is located: genes with low mutation rates embedded in gene poor regions lose approximately 17% of their adaptive substitutions whereas genes with high mutation rates embedded in gene rich regions lose approximately 60%. Hence, we have shown evidences that recombination, mutation, and gene density are important determinants of the rate of adaptive evolution within the *Drosophila* genome.

## Results

To investigate the role of recombination, mutation, and gene density in determining the rate of adaptive evolution, we compiled 6,141 autosomal protein-coding genes from *Drosophila* for which we have polymorphism data from *D. melanogaster* and divergence out to *D. yakuba.* For most of our analyses, we use polymorphism data from the *D. melanogaster* Genetic Reference Panel (DGRP) which was sampled from Raleigh, North Carolina (Mackay et al. 2012). However, in some analyses we compare our results to those obtained using the flies sampled from Gikongoro, Rwanda (DPGP2, Pool et al. 2012)*.* To estimate the rate of adaptive evolution we use the DFE-alpha method (Eyre-Walker and Keightley 2009), a derivative of the McDonald–Kreitman test (McDonald and Kreitman 1991) which corrects for slightly deleterious mutations. In this method it is assumed that mutations at one set of sites (in this analysis synonymous sites) are neutral and that selection acts upon the mutations at another set of sites (nonsynonymous sites). The site frequency spectra (SFS) of synonymous and nonsynonymous polymorphisms are used to infer the distribution of fitness effects (DFEs) of neutral and deleterious mutations at the nonsynonymous sites and this information is used, in conjunction with the level of synonymous divergence, to predict how many neutral and nearly neutral nonsynonymous substitutions are expected. If the observed divergence at nonsynonymous sites exceeds this expectation, adaptive evolution is inferred and quantified. The rate of adaptive evolution is typically estimated using one of three statistics: α, the proportion of substitutions that are adaptive, ω_A_, the rate of adaptive evolution relative to the mutation rate, and *K_a_*_+_, the rate of adaptive amino acid substitution, which is equal to α*K_a_.* The α statistic conflates the rates of adaptive and nonadaptive substitution and hence is not useful for our purposes here, and ω_A_ is not useful for studying the effects of mutation on the rate of adaptive evolution because it controls for the factor being investigated, hence we have investigated how *K_a_*_+_ depends upon the rate of recombination, mutation, and gene density. However, in terms of recombination and gene density, we get qualitatively similar results whether we use *K_a_*_+_ or ω_A_.

### Recombination and Adaptation

**Fig. 1.**
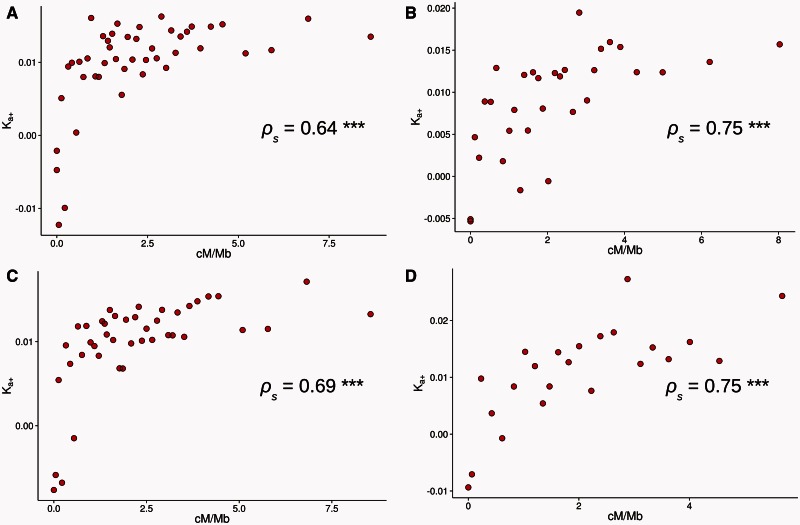
Relations between *K_a_*_+_ in the *y* axis and the rate of recombination (cM/Mb) in the *x* axis: (*A*) using DGRP polymorphism data, North Carolina population, (*B*) using DPGP2 polymorphism data, Rwanda population, (*C*) excluding immune response and testes related genes, and (*D*) using short intron sites as neutral reference (< 66 nt, bases from 8 to 30). Each data point has been estimated binning genes. The number of genes, the average recombination rate and *K_a_*_+_ estimate for each bin can be consulted in the supplementary table S1, Supplementary Material online. *ρ*_s_: Spearman’s rank correlation coefficient, with significance denoted by asterisks (***<0.001; **<0.01; *<0.05).

We first studied the relationship between recombination rate and *K_a_*_+_. To estimate the rate of adaptive evolution, it is necessary to combine data from several genes because estimates tend to be error prone and sometimes undefined for individual genes. We therefore grouped genes into 45 bins of 136 genes each based on their rates of recombination. The results are shown in figure 1*A.* There is a highly significant positive relationship between the rate of adaptation and the recombination rate (Spearman’s rank correlation coefficient *ρ*_s_ = 0.64, *P* < 0.001). However, for values beyond approximately 2 cM/Mb the relationship between recombination and adaptation reaches an asymptotic value. We interpreted the asymptote greater than 2cM/Mb as the rate of adaptive evolution that would occur if there was no effective HRi upon advantageous mutations. In order to test whether a curvilinear relationship fits the data better than a linear model, we fit the function *y = a + b ·e ^−^^cx^* to our data and compared it to the fit of a linear model (see supplementary figs. S1*A* and *B*, Supplementary Material online). Table 1 shows the inferred parameters, the *R*^2^ and AIC values for the two models. In terms of both AIC and *R*^2^ the curvilinear model is favored.

**Table 1.** Linear and Curvilinear Fit Inferred Parameters, *R*^2^ and AIC for Several Data Sets Where *y* = *K_a_*_+_ and *x* = cM/Mb.

	*n*	Linear (*y* ∼ *a* + *b* · *x*)				Curvilinear (*y* ∼ *a* + *b* · *e* − *cx*)					Pr(>F)
		*a*	*b*	*R*^2^	AIC	*a*	*b*	*c*	*R*^2^	AIC	
DGRP	45	0.0057	0.0018	0.27	−336.85	0.0126	−0.0186	2.1237	0.67	−369.78	1.36E−08
DGRP	11	0.0058	0.0019	0.52	−88.58	0.0132	−0.0144	1.4382	0.94	−110.07	0.00005617
DPGP2	31	0.0042	0.0021	0.38	−236.78	0.0148	−0.0148	0.5843	0.51	−241.75	0.01286
DPGP2	11	0.0036	0.0024	0.54	−84.45	0.0144	−0.0156	0.7429	0.72	−88.00	0.0511
w/o IT	42	0.0059	0.0017	0.32	−327.03	0.0124	−0.0183	2.0991	0.69	−358.04	3.723E−08
High Fop	15	0.0026	0.0017	0.51	−125.34	0.0096	−0.0144	1.2983	0.91	−148.41	0.00001086
Med Fop	15	0.0081	0.0009	0.63	−150.09	0.0135	−0.0069	0.4013	0.72	−151.74	0.09402
Low Fop	15	0.0037	0.0033	0.43	−101.74	0.0161	−0.0269	1.7241	0.80	−115.49	0.0004943
Short Int	23	0.0026	0.0040	0.51	−164.96	0.0175	−0.0229	0.9801	0.67	−171.86	0.005978
GenH-MutH	12	0.0009	0.0033	0.55	−86.69	0.0193	−0.0222	0.4157	0.62	−86.55	0.2504
GenH-MutL	12	0.0030	0.0016	0.26	−91.74	0.0078	−0.1039	18.68	0.90	−114.09	0.00002975
GenL-MutH	12	0.0077	0.0031	0.37	−76.76	0.0219	−0.0280	1.2229	0.84	−91.25	0.0005979
GenL-MutL	12	0.0054	0.0009	0.27	−99.33	0.0095	−0.0128	2.0985	0.87	−118.20	0.0001117

Note.—The first column (*n*) is the number data points (or bins). The *P* value from the *F*-test used to compare the linear and curvilinear fit is in the last column. The DGRP data set is in rows 1–2. The DPGP2 data set is in rows 3–4. In row 5 (w/o IT) we excluded immune response and testes related genes. Rows 6–8 show the results for high, medium and low *Fop* genes, respectively. Row 9 (Short Int) shows the results using short intron sites (<66, bases 8–30) as neutral reference. Rows 10–13 show the results for GenH-MutH, GenH-MutL, GenL-MutH, and GenL-MutL genes, respectively.

Our results are in contrast to those of Campos et al. (2014) who found that ω_A_ was linearly related to the rate of recombination. The difference between the two analyses could be due to the fact that we have used a different measure of adaptive evolution, to differences in the number of bins, to differences in the populations from which the polymorphism data were derived, or finally to differences in the way in which the rate of recombination was estimated. The difference between the two studies is not due to the measure of adaptive evolution used since we observe a curvilinear relationship using both *K_a_*_+_ and ω_A_ (see fig. 1*A* and supplementary fig. S2*A*, Supplementary Material online). The number of bins, or the binning strategy, does not seem a plausible explanation either because when we use the binning strategy used by Campos et al. (2014) (ten bins above 0 cM/Mb and one bin with 0 cM/Mb) we again observe a highly significant curvilinear relation (see supplementary fig. S3*A* and *B* and table S1, Supplementary Material online). Campos et al. (2014) used two different estimates of recombination rate: one based on low resolution visible markers (Fiston-Lavier et al. 2010), the other one on the high resolution recombination map obtained by Comeron et al. (2012) using single nucleotide polymorphism markers. For both data sets, Campos et al. (2014) observed a linear relationship. However, instead of taking point estimates of the recombination rate from the Comeron et al.’s high resolution map, as we have done, Campos et al. (2014) fitted a LOESS regression to the data which smoothes out the original high resolution recombination map. We have repeated the correlation analysis of Campos et al. using their polymorphism (from Gikongoro, Rwanda [DPGP2, Pool et al. 2012]) and divergence genomic data together with the original unsmoothed high resolution recombination map. In contrast to the linear relationship they originally reported we found the same highly significant curvilinear pattern that we observed using the DGRP polymorphism data (see fig. 1*B*, supplementary fig. S3*C* and *D* and table S1, Supplementary Material online) (*ρ*_s_ = 0.75, *P* < 0.001). Thus, the linear relationship between the rate of adaptive evolution and the rate of recombination observed by Campos et al. seems to be a consequence of smoothing the recombination rate estimates rather than differences in the adaptive evolution statistics, the binning strategy, or differences in the populations from which the polymorphism data were derived. For this reason, all subsequent analyses presented here are based on the DGRP data from Raleigh, North Carolina (Mackay et al. 2012), because these data have greater coverage and number of sampled chromosomes. This result is important for future studies seeking to quantify the consequences of HRi. Here, we show the benefit of using high-resolution recombination maps relative to “smoothed” or low resolution maps which may generate biased/imperfect results and conclusions, at least in *Drosophila.*

The positive correlation between recombination rate and *K_a_*_+_ could be due to a number of potential biases in the data set. If recombination is mutagenic we would expect a positive correlation between *K_a_*_+_ and the rate of recombination. However, previous analyses have found no evidence to suggest that recombination is mutagenic (Begun and Aquadro 1992; Begun et al. 2007; McGaugh et al. 2012) and we find no correlation between the rate of substitution in short introns, which are believed to be the most neutral class of sites, and the rate of recombination (*ρ*_s_ = 0.01, *P* = 0.89). Furthermore, we find a positive curvilinear relationship between ω_A_, which is *K_a_*_+_ divided by our estimate of the mutation rate, *K*_4_, and the rate of recombination (see supplementary fig. S2, Supplementary Material online).

An artifactual positive correlation between the rate of adaptive evolution and the rate of recombination could also be caused if some classes of genes with high rates of adaptation are preferentially located in regions with high rates of recombination. There is evidence that immune system (Obbard et al. 2009) and male-biased or testes specific (Pröschel et al. 2006; Haerty et al. 2007) genes undergo higher rates of adaptive evolution than other genes. We confirm this result taking into account the influence of slightly deleterious mutations, which the previous analyses did not (see supplementary fig. S4, Supplementary Material online). We find that immune and testes-specific genes together exhibit adaptive rates 1.37 × faster than other genes (the difference between immune/testes specific genes and other “control” genes is significant as judged by a permutation test, *P* = 0.017). However, if we remove immune and testes specific genes we still observe a highly significant curvilinear correlation between recombination rate and *K_a_*_+_ (see fig. 1*C* and table 1) (*ρ*_s_ = 0.69, *P* < 0.001).

In estimating the rate of adaptive evolution we have assumed that synonymous mutations are neutral, however selection is known to act upon synonymous sites in *Drosophila* (reviewed by Hershberg and Petrov 2008). In many cases, this is thought to be a result of selection favoring codons that can be translated more rapidly or accurately (Shields et al. 1988; Akashi 1994, 1995; Carlini and Stephan 2003; Stoletzki and Eyre-Walker 2006). Additionally, synonymous sites may be under selection to maintain (or avoid) splicing enhancers (Parmley et al. 2006), messenger RNA secondary structures (Parsch et al. 1997; Baines et al. 2004; Stoletzki 2008) or particular short sequence motifs (Antezana and Kreitman 1999). Lawrie et al. (2013) have shown that approximately 22% of all 4-fold synonymous sites in *D. melanogaster* are under strong purifying selection, although the specific functional mechanism underlying this strong constraint is unknown. Consistent with weak selection favoring codons that have to be translated more rapidly or accurately we confirm previous results that *K*_4_ is significantly correlated to a measure of codon usage bias, *Fop* (the frequency of optimal codons) (*ρ*_s_ =−0.4, *P* < 0.001) (Sharp and Li 1987, 1989; Moriyama and Hartl 1993; Bierne and Eyre-Walker 2003, 2006). However, we expect that any sort of weak selection on synonymous mutations would generate a positive correlation between *K_a_*_+_ and recombination rate. This is because we expect that genes located in regions of high recombination, where selection on synonymous sites is more efficient (Kliman and Hey 1993; Haddrill et al. 2007; Campos et al. 2012), will tend to have a higher estimate of *K_a_*_+_ because weak negative selection on synonymous mutations inflates the number of synonymous polymorphisms relative to the number synonymous substitutions. Therefore, to investigate whether selection on synonymous codon usage affects our adaptation estimates we divided our genes into three roughly equal groups according to their *Fop* value, and within each of these *Fop* groups we divided the data into 15 groups of genes according to their recombination rate. We observe the same highly significant curvilinear relationship within each of the 3 *Fop* categories (see supplementary fig. S5 and table S1, Supplementary Material online) (for high *Fop* genes *ρ*_s_ = 0.87, *P* < 0.001; medium *Fop* genes *ρ*_s_ = 0.78, *P* < 0.001; low *Fop* genes *ρ*_s_ = 0.76, *P* < 0.001). We also repeated our analysis using a smaller data set of 3,369 genes where we can use polymorphisms and substitutions in short introns (<66 bp) as the neutral standard. This data set is smaller because not all genes fulfil the intron quality and length criteria (see Materials and Methods). The same curvilinear pattern is observed (see fig. 1*D* and table 1) and the strength of the correlation is equivalent to that found with 4-fold sites (*ρ*_s_ = 0.75, *P* < 0.001). Hence, selection on codon usage does not seem to be responsible for the shape or the strength of the relationship between the rates of adaptive evolution and recombination.

### Gene Density and Adaptation

**Fig. 2.**
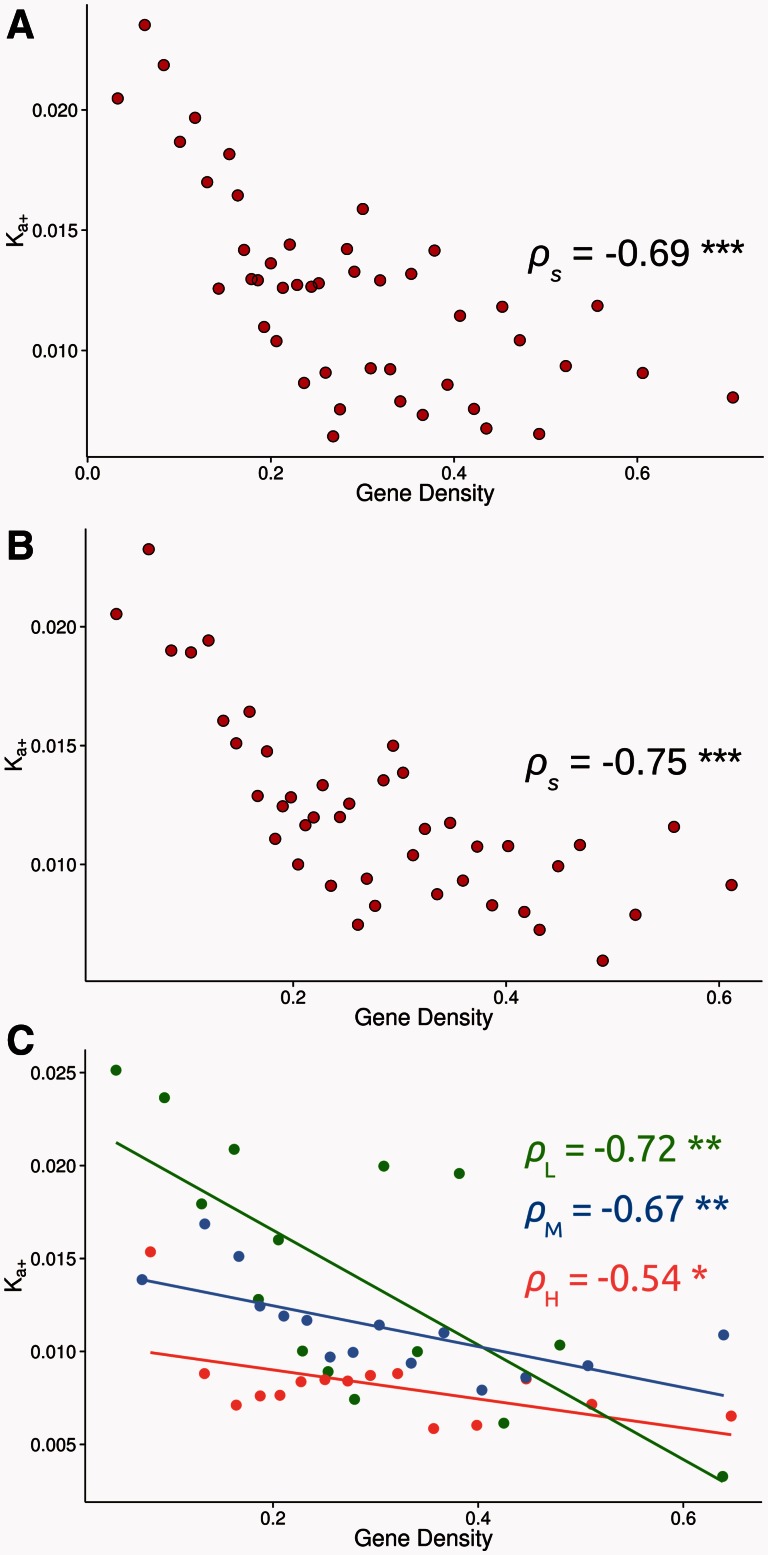
Relationship between *K_a_*_+_ in the *y* axis and the gene density (coding sequences sites /100,000 sites) in the *x* axis: (*A*) using the whole data set, (*B*) excluding immune response and testes related genes and (*C*) splitting the data set into three *Fop* groups. Genes belonging to the high (H) group are in red, medium (M) genes are in blue, and low (L) genes are in green. Each data point has been estimated binning genes. The number of genes, the average gene density, *Fop*, and *K_a_*_+_ estimate for each bin can be consulted in the supplementary table S4, Supplementary Material online. *ρ*_s_: Spearman’s rank correlation coefficient, with significance denoted by asterisks (***<0.001; **<0.01; *<0.05). The lines are least-squares regressions but should be regarded only as indicative, in view of the binning of the data.

The strength of HRi is expected to depend on both the rate of recombination and the density of selected sites across the genome. We might therefore expect a negative correlation between the rate of adaptive evolution and gene density, a relationship we observe (see fig. 2*A*) (*ρ*_s_ = −0.69, *P* < 0.001). The highly significant correlation remains if we exclude immune and testes specific genes (see fig. 2*B*) (*ρ*_s_ = −0.75, *P* < 0.001). However, contrary to expectations under HRi, we find, as Hey and Kliman (2002) did, that there is a weak positive correlation between codon usage bias, as measured by *Fop* and gene density (*ρ*_s_ = 0.07 and *P* < 0.001). To check that this positive correlation was not inducing an artifactual negative correlation between *K_a_*_+_ and gene density we divided our genes into three categories according to *Fop* and repeated our analysis. In all three groups we observe a highly significant negative correlation between *K_a_*_+_ and gene density (see fig. 2*C*) (high *Fop* genes *ρ*_s_ = −0.54, *P* < 0.05; medium *Fop* genes *ρ*_s_ = −0.67, *P* < 0.01; low *Fop* genes *ρ*_s_ = −0.72, *P* < 0.01). Qualitatively similar results are obtained between ω_A_ and gene density (see supplementary fig. S6, Supplementary Material online).

### Mutation and Adaptation

To investigate whether the rate of adaptive evolution is correlated to the mutation rate is not straightforward because we need to use the rate of synonymous substitution to estimate both the mutation rate and the rate of adaptive evolution. This lack of statistical independence between estimates will tend to generate a negative correlation between *K_a_*_+_ and *K*_4_ just through sampling error. To avoid problems of nonindependence we split our synonymous substitution estimate, *K*_4_, into three independent variables by sampling from a hypergeometric distribution (see Materials and Methods: Hypergeometric Sampling); we used *K*_4,1_ to rank genes and assign genes to bins, *K*_4,2_ to estimate the rate of adaptive evolution*,* and *K*_4,3_ as an estimate of mutation rate for each bin*.*

**Fig. 3.**
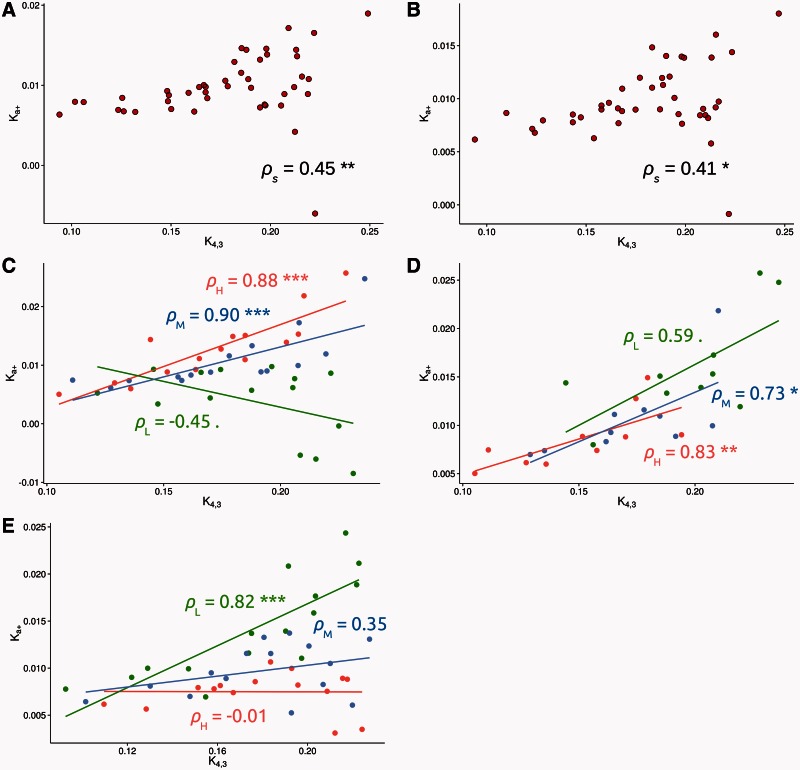
Relationship between *K_a_*_+_ in the *y* axis and an estimate of the mutation rate (*K*_4,3_) in the *x* axis: (*A*) using the whole data set, (*B*) excluding immune response and testes related genes, (*C*) splitting the data set into three recombination groups, (*D*) splitting the data set into three *Fop* groups after removing low recombination rate genes (<1.32 cM/Mb), and (*E*) splitting the data set intro three gene density groups. Genes belonging to the high (H) group are in red, medium (M) genes are in blue, and low (L) genes are in green. Each data point has been estimated binning genes. The number of genes, the average mutation rate (*K*_4,3_), recombination rate, gene density, *Fop*, and *K_a_*_+_ estimate for each bin can be consulted in the supplementary table S5, Supplementary Material online. *ρ*_s_: Spearman’s rank correlation coefficient, with significance denoted by asterisks (***<0.001; **<0.01; *<0.05; 0.1–0.05). The lines are least-squares regressions but should be regarded only as indicative, in view of the binning of the data.

The data were divided, as with the recombination rate analyses, into 45 mutation bins of 136 genes each, but this time the data were divided according to their *K*_4,1_ value. Doing this we found a highly significant positive correlation between *K_a_*_+_ and *K*_4,3_ (*ρ*_s_ = 0.45, *P* < 0.001) (see fig. 3*A*). As with the correlation between *K_a_*_+_ and the rate of recombination, this correlation could be spurious due to several sources of bias. The correlation is still highly significant even if we exclude testes and immune system related genes (*ρ*_s_ = 0.41, *P* < 0.01) (see fig. 3*B*), suggesting that the correlation between *K_a_*_+_ and *K*_4,3_ is not a consequence of the nonrandom distribution of this kind of genes relative to the mutation rate. Natural selection on codon usage is expected to weaken rather than generate an artifactual positive correlation between *K_a_*_+_ and *K*_4,3_, because selection on codon usage should reduce the rate of synonymous substitutions more than the level of synonymous polymorphism. To investigate whether selection on codon usage has an effect on the relationship between *K_a_*_+_ and *K*_4,3,_ we divided the data set into three recombination rate levels and three *Fop* levels, and within each recombination rate and *Fop* class we grouped the genes into five groups according to their mutation rate (this yielded 45 bins of 136 genes each). We separate the data according to their recombination rate because it affects both the rate of adaptive evolution as well as the efficiency of selection on codon usage. The correlation between *K_a_*_+_ and *K*_4,3_, for each recombination rate and *Fop* category is shown in figure 3*C* and *D*, respectively. The graphs suggest that selection on codon bias makes little difference to the correlation between *K_a_*_+_ and *K*_4,3_, but that the relationship is strongly affected by the rate of recombination; this is not surprising because we have shown above that genes with low rates of recombination undergo very little adaptive evolution (see fig. 1) and are therefore not likely to be influenced by the rate of mutation. To investigate this more formally we performed an analysis of covariance (ANCOVA), grouping genes by their *Fop* and recombination rate levels. In ANCOVA, a set of parallel lines are fitted to the data, one for each group. This enables a test of whether the common slope of these lines is significantly different from zero, and one can also investigate whether the groups differ in the dependent variable for a given value of the independent variable by testing whether the lines have different intercepts and slopes. If we consider *Fop* and recombination rate as fixed factors we find no significant correlation between *K_a_*_+_ and *K*_4,3_ (ANCOVA *P* = 0.16). However, we find evidence that the slopes (ANCOVA *P* < 0.001) and intercepts (ANCOVA *P* < 0.001) differ between recombination rate categories, but there is no evidence that either the slope or intercept differs between *Fop* categories. If genes with low recombination rates (from 0 to 1.32 cM/Mb) are excluded, a very strong positive correlation between *K_a_*_+_ and *K*_4,3_ is found for the rest of the data set (see supplementary fig. S7, Supplementary Material online) (*ρ*_s_ = 0.82 and *P* < 0.001). There is no evidence within this data set that the slope or intercept differ according to rate of recombination or the level of codon bias. As an alternative approach to controlling the effect of selection on codon usage on our estimates of the mutation rate, we regressed *K*_4,3_ against the rate of recombination and *Fop* and used the residuals as a measure of the mutation rate. We find a strong positive correlation between *K_a_*_+_ and the residuals (see supplementary fig. S8, Supplementary Material online) (*ρ*_s_ = 0.42 and *P* < 0.01), suggesting that the correlation between *K_a_*_+_ and *K*_4,3_ is not a result of weak selection on 4-fold sites.

We observe that the rate of recombination affects the relationship between *K_a_*_+_ and *K*_4,3_ so we might also expect gene density to have a similar effect—for the relationship between the rate of adaptive evolution and the mutation rate to be stronger in regions of the genome with lower gene density. To investigate this we divided the data set into three gene density levels, and within each gene density group, we grouped the genes into 15 bins according to their mutation rate (this yielded 45 bins of 136 genes each). Figure 3*E* shows the relationship between the mutation rate and the rate of adaptation for each gene density group. Again we performed an ANCOVA grouping genes by its gene density. If we consider gene density as a fixed factor we find a significant correlation between *K_a_*_+_ and *K*_4,3_ (ANCOVA *P* < 0.01). However, we find that the slopes (ANCOVA *P* < 0.01) and intercepts (ANCOVA *P* < 0.05) differ between gene density categories. When low gene density genes are excluded we find no significant correlation between *K_a_*_+_and *K*_4,3_ (ANCOVA *P* = 0.51) and no evidences for differences in the slopes (ANCOVA *P* = 0.70) or intercepts (ANCOVA *P* = 0.13) between medium and high gene density groups. Thus, we only observe a highly significant positive correlation between *K_a_*_+_ and *K*_4,3_ for the low gene density genes (*ρ*_s_ = 0.82, *P* < 0.001) and a nonsignificant positive correlation for the rest of gene density categories (see fig. 3*E*).

Altogether our results show that genome-wide there is a significant and positive relation between the mutation rate and the rate of adaptation (see fig. 3*A* and *B* and supplementary figs. S7 and S8, Supplementary Material online), because genes with higher rates of mutation are more likely to produce the genetic variation needed for adaptation. Nonetheless, this does not necessarily mean that this positive correlation holds for the whole genome. In fact, the strength and sign of the relationship depends on the rate of recombination (see fig. 3*C*) and the gene density (see fig. 3*E*). We have shown that when the gene density is high and/or the recombination rate is low there is little correlation between the mutation rate and the rate of adaptation due to HRi.

### The Proportion of Adaptive Substitutions Lost to HRi

Our results show that the rate of adaptive evolution is significantly impeded in low recombining and gene dense regions of the *Drosophila* genome. But how many adaptive substitutions are lost because of HRi? And how does the mutation rate and the gene density affect the intensity of the HRi? To answer these questions we fit a LOESS curve to the relationship between *K_a_*_+_ and recombination rate, which clearly approaches an asymptote above 2 cM/MB (see supplementary fig. S1*C*, Supplementary Material online). The asymptote greater than 2cM/Mb can be interpreted as the rate of adaptive evolution that would occur if there was no HRi. The LOESS curve decreases below the asymptotic value as the rate of recombination decreases, and the difference between the asymptote and the LOESS curve can be interpreted as the number of adaptive substitutions that are lost due to HRi. Using this approach we estimate, after weighting by the number of sites involved that 27.2% (95% confidence intervals [CIs] obtained by bootstrapping by gene [20.6%, 33.8%]) of all adaptive amino acid substitutions that would be fixed in an effectively free recombining genome are lost because of HRi. Here, we call this proportion of adaptive substitutions lost to HRi as the *f*_HRi_. Some of the estimates of *K_a_*_+_ inferred from the LOESS curve are negative; however, even our estimate of the proportion of adaptive substitutions lost to HRi is largely unchanged even if we set these to zero: 27.1% (95% CIs [20.6%, 33.2%]).

**Fig. 4.**
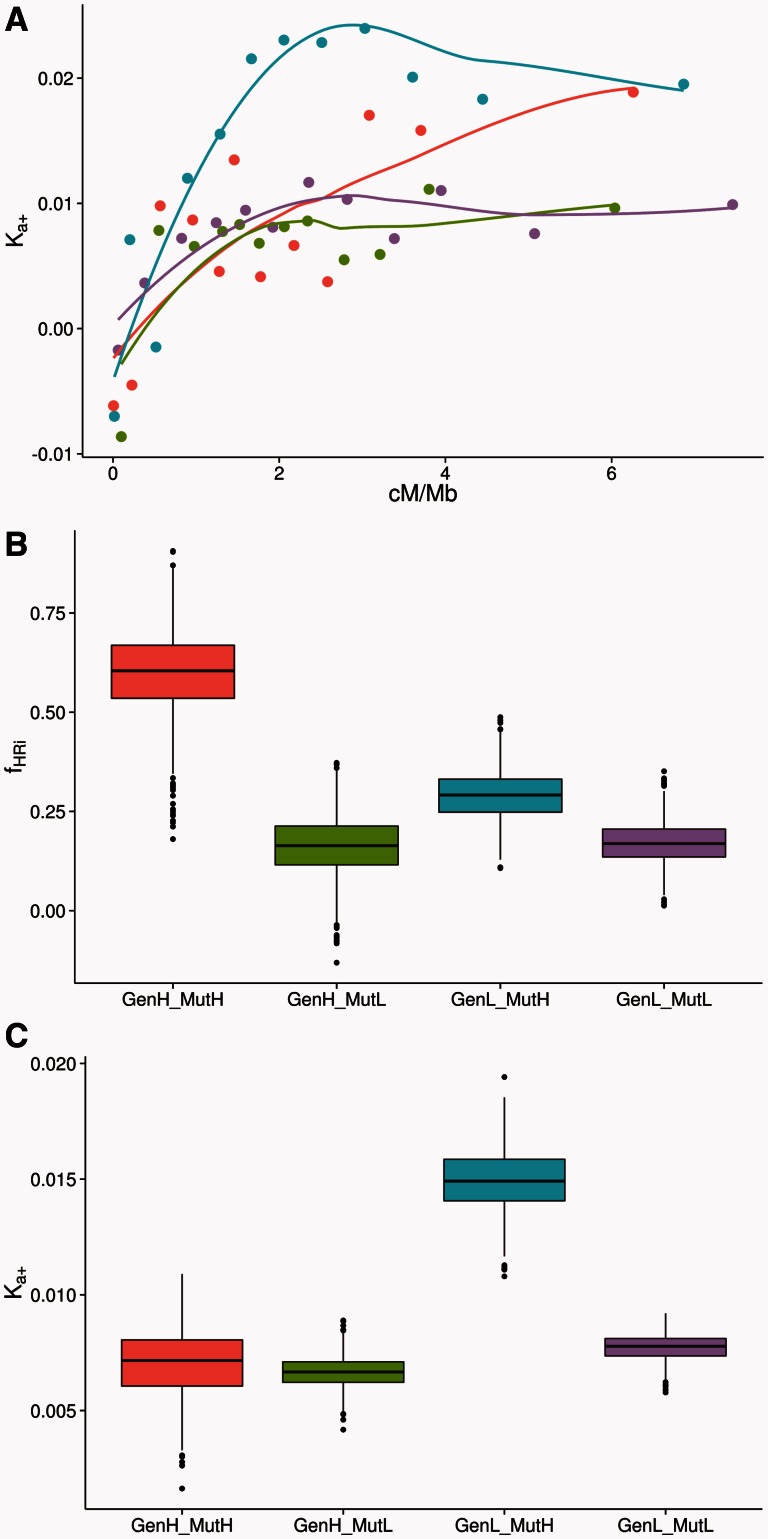
(*A*) Relationship between *K_a_*_+_ in the *y* axis and the rate of recombination (cM/Mb) in the *x* axis for each gene category; the lines are LOESS regressions. (*B*) Bootstrap *f*_HRi_ values and (*C*) bootstrap *K_a_*_+_ values for each gene category, respectively. Each data point has been estimated binning 128 genes according to their gene density, mutation rate (*K*_4,1_) and recombination rate (in cM/Mb). GenH-MutH genes are in red, GenH-MutL genes are in green, GenL-MutH genes are in blue, and GenL-MutL genes are in purple; see the full description of these gene categories in the main text.

However*,* HRi is expected to be more prevalent in loci with higher rates of mutation and/or in loci located in gene rich regions, because this will increase the chance that a selected mutation will be segregating with other mutations subject to selection. To investigate whether this is the case in *Drosophila,* we repeated the analysis above splitting the data set into different categories according to a gene's mutation rate and the gene density of the window where the gene is located. First, we divided the data set into two according to the gene density, and within each gene density group we did two equally sized groups according to gene’s mutation rate. Qualitatively similar results are obtained if we split first by the mutation rate and then by the gene density (data not shown). To split by the mutation rate, we split first *K*_4_ into two independent variates by sampling from a hypergeometric distribution. *K*_4,1_ was used to divide the genes into different mutation rate categories, while *K*_4,2_ was used to calculate *K_a_*_+_. In this way we ensured that the estimates of adaptive evolution were not influenced by the way in which the data was divided. For simplicity we labeled these four groups in the following way: GenH-MutH (high gene density and high mutation rate genes), GenH-MutL (high gene density and low mutation rate genes), GenL-MutH (low gene density and high mutation rate genes), and GenL-MutL (low gene density and low mutation rate genes). The relationship between *K_a_*_+_ and recombination rate for each gene category can be seen in figure 4*A.* The strength of the relationship is equivalent to that found previously for the entire data set (GenH-MutH genes *ρ*_s_ = 0.67, *P* < 0.05; GenH-MutL genes *ρ*_s_ = 0.48, *P* < 0.05; GenL-MutH genes *ρ*_s_ = 0.67, *P* < 0.05; and GenL-MutL genes *ρ*_s_ = 0.55, *P* < 0.05). However, the relationship appears to be approximately linear for GenH-MutH, whereas for the other categories it is significantly curvilinear (see table 1).

Because the GenH-MutH genes show no asymptote we can only attempt to estimate a lower bound on the proportion of substitutions lost to HRi; we take the value of *K_a_*_+_ for genes above 5cM/MB as our estimate of the rate of adaptation without HRi; for the other categories we use the 2cM/MB threshold as before. Using these thresholds we find that the proportion of substitutions lost to HRi differs significantly between groups of genes; genes with high mutation rates in gene dense regions lose significantly more substitutions than genes in other categories (GenH-MutH vs. GenH-MutL bootstrap *P* < 0.01, GenL-MutH bootstrap *P* < 0.05, GenL-MutL bootstrap *P* < 0.01), which are not significantly different to each other. GenH-MutH genes are estimated to have lost approximately 59.7% (95% CIs [41.5%, 75.6%]) of all substitutions due to HRi compared with approximately 20% in the other categories (see fig. 4*B* and table 2). If we calculate the overall loss of substitutions to HRi combining the data from the four categories we estimate approximately 35.9% (95% CIs [27.0%, 44.2%]) of all adaptive amino acid substitutions that would be fixed in an effectively free recombining genome have been lost because of HRi. In any case, this new estimate of the overall *f*_HRi_ is not significantly higher than the previous estimate which was approximately 27% (bootstrap *P* value = 0.18).

**Table 2.** Bootstrap Mean and 95% CIs for Several Summary Statistics.

Category	*K*_a+_	*K*^4,2^	f_HRi_	AA+ (kb)	Lost AA+ (kb)	Global *f*_HRi_
GenH-MutH	0.0070 (0.0045, 0.0092)	0.24 (0.23, 0.25)	0.60 (0.41, 0.76)	9.97 (6.37, 13.07)	15.46 (7.74, 22.77)	0.36 (0.27, 0.44)
GenH-MutL	0.0067 (0.0054, 0.0079)	0.18 (0.17, 0.18)	0.16 (0.02, 0.28)	9.58 (7.83, 11.34)	1.87 (0.26, 3.37)	
GenL-MutH	0.0149 (0.0128, 0.0171)	0.24 (0.23, 0.24)	0.29 (0.19, 0.40)	18.93 (16.17, 21.69)	7.83 (4.80, 11.17)	
GenL-MutL	0.0077 (0.0067, 0.0086)	0.17 (0.16, 0.17)	0.17 (0.08, 0.27)	9.50 (8.29, 10.62)	1.97 (0.88, 3.19)	

Note.—*K_a_*_+_, adaptive nonsynonymous substitution rate per site (corrected by Jukes and Cantor 1969 method); *K*_4,2_, 4-fold substitution rate per site (corrected by Tamura 1992 method); *f*_HRi_, fraction of lost adaptive substitutions to HRi; AA+ (kb), absolute number of adaptive amino acid mutations that were fixed (in kb); lost AA+ (kb) absolute number of adaptive amino acid mutations that would be fixed in the absence of HRi (in kb) and Global *f*_HRi_, overall fraction of lost adaptive substitutions to HRi.

Finally, although there is variation in the fraction of advantageous mutations lost to HRi across gene categories, how many adaptive substitutions do they fix? Figure 4*C* shows the boxplots of the average *K_a_*_+_ for each gene category across the bootstrap replicates. The genes with the highest adaptation rates are those with high mutation rates located in gene poor regions (GenL-MutH genes) *K_a_*_+_ = 0.0149 (95% CIs [0.0128, 0.0171]), whereas the rest of gene categories show similar levels of adaptation: GenH-MutH genes *K_a_*_+_ = 0.007 (95% CIs [0.0044, 0.0092]), GenH-MutL genes *K_a_*_+_ = 0.0067 (95% CIs [0.0054, 0.0069]), and GenL-MutL genes *K_a_*_+_ = 0.0077 (95% CIs [0.0067, 0.0086]). So although GenH-MutH and GenL-MutH genes have a significantly higher mutation rate than GenH-MutL and GenL-MutL genes (*K*_4,2_ fold-change = 1.4, bootstrap *P* < 0.001), GenH-MutH genes lose many more substitutions to HRi than low mutation rate genes (GenH-MutL and GenL-MutL genes) (see the statistics above), and as a consequence the rate of adaptive evolution ends up being similar for GenH-MutH, GenH-MutL, and GenL-MutL genes (GenH-MutH vs. GenH-MutL bootstrap *P* = 0.41; GenH-MutH vs. GenL-MutL bootstrap *P* = 0.35; GenH-MutL vs. GenL-MutL bootstrap *P* = 0.13). In contrast, GenL-MutH genes are less prone to HRi due to their low gene density and so they can adapt faster than any other gene category due to their higher mutation rates (GenL-MutH vs. GenH-MutH bootstrap *P* < 0.001, GenH-MutL bootstrap *P* < 0.001, GenL-MutL bootstrap *P* < 0.001).

## Discussion

We have shown that the rate of adaptive protein evolution is positively correlated to both the rate of recombination and the mutation rate, whereas it is negatively correlated to the gene density in *D. melanogaster.* We have shown that these correlations are not due to an enrichment of immune response and testes related genes in regions of low gene density or in regions of high recombination or mutation, or due to selection on synonymous sites. Instead it seems likely that the rate of adaptive evolution is positively correlated to the rate of recombination and negatively correlated to the gene density because of HRi and that it is positively correlated to the rate of mutation because genes with higher rates of mutation are more likely to produce the genetic variation needed for adaptation. Interestingly, the positive correlation between the rate of adaptation and the mutation rate disappears for genes located in regions of low recombination or in rich gene regions confirming that HRi is more prevalent when the number of selected mutations is high and the genetic distance among them is small. This work quantifies for the first time the global impact of HRi on a given genome. We estimate that approximately 27% of all adaptive mutations, which would go to fixation if there was free recombination, are lost due to HRi. We show that this estimate depends upon the mutation rate and the gene density with genes with high mutation rates located in gene rich regions losing a greater proportion of their adaptive substitutions to HRi (∼60%) than genes with low mutation rates located in poor gene regions (∼17%).

The recombination rate data we have used only includes cross-overs (CO) and excludes gene conversion (GC) events. This is because GC is expected to be a much less important force reducing HRi than CO. Although GC events occur approximately 5 times more frequently than COs (Comeron et al. 2012), the GC tract lengths are quite short at about 500 bp (Comeron et al. 2012) and hence lead to relatively little recombination. The fact that GC is largely ineffective in reducing HRi can be inferred from the presence of HRi in regions of the genome with very low rates of CO, because even these regions have moderate levels of GC—the frequency of GC varies little across the *Drosophila* genome (Comeron et al. 2012).

An open question is to what extent HRi affects rates of adaptive evolution in other species. The strength of HRi depends on the rate of mutation at selective sites, the DFEs and the rate of recombination; the greater the density of selected mutations per map unit, and the more strongly selected they are, the greater the effect of HRi will be on weakly selected mutations. Is HRi likely to be an important force in a species like humans? Humans are estimated to have a genomic rate of harmful mutation of 2.1 (Lesecque et al. 2012) that is approximately twice that in *Drosophila* at 1.2 (Haag-Liautard et al. 2007), and although, the human genome is approximately 20 × greater in size than the *Drosophila* genome, linkage disequilibrium declines approximately 500 × more slowly in humans than *Drosophila.* Taken together these results suggest that HRi, at least from deleterious mutations, might be more important in humans than *Drosophila.* However, this needs to be confirmed by analysis, and this is difficult because humans appear to have undergone relatively little adaptive evolution (Boyko et al. 2008; Eyre-Walker and Keightley 2009; Gossmann et al. 2012) and this makes analysing the factors that affect the rate of adaptive evolution difficult. The potentially higher level of HRi in humans may explain in part why our species appears to have undergone relatively little adaptive evolution compared with *Drosophila* (Gossmann et al. 2012). However, the effect of HRi will depend upon the distributions of fitness effects and this is something we have limited information about in both of these species. It will be of great interest to do similar analyses to those performed here in other species.

The loss of adaptive substitutions to HRi can potentially tell us something important about the strength of selection acting on some advantageous mutations, since weakly selected mutations are those that are most likely to be affected by HRi (McVean and Charlesworth 2000; Comeron and Kreitman 2002; Comeron et al. 2008). This will require further analysis and population genetic modeling, but in combination with other sources of information, for example, the dip in diversity around nonsynonymous substitutions (Sella et al. 2009), the SFS (Schneider et al. 2011), and the high frequency variants that are left by selective sweeps (Fay and Wu 2000), and it may be possible to infer much more about the DFE of advantageous mutations than previously thought.

The fact that so many adaptive substitutions are lost to HRi begs the question why *Drosophila* does not have a higher rate of recombination, particularly in areas where there is little or no recombination in its genome. This may be because selection on modifiers of the recombination rate is weak; a modifier that elevates the rate of recombination may allow advantageous mutations to spread more easily, but by its very nature it will tend to disassociate itself from the advantageous mutations that it helps spread. It therefore gets little or no benefit from the positive effects it causes. Another interesting question is why genes do not move from low recombination rate regions. This is probably because they only get an advantage from moving if there is an advantageous mutation spreading through the population at that gene when the gene translocates, or shortly after it has translocated.

## Conclusions

Our analysis has shown how the rate of recombination, the mutation rate, and gene density affect the rate of adaptation within the *D. melanogaster* genome. We find that the rate of adaptive amino acid substitution is positively correlated to both recombination rate and an estimate of the mutation rate, while it is negatively correlated to gene density. We also find that this correlation is robust to controlling for each other, synonymous codon bias and gene functions related to immune response and testes. Finally we estimate that on average at least ∼27% of all advantageous substitutions have been lost because of HRi and that this quantity depends gene’s mutation rate and the gene density where the gene is located: genes with low mutation rates embedded in gene poor regions lose approximately 17% of their adaptive substitutions whereas genes with high mutation rates embedded in gene rich regions lose approximately 60%. Hence, we have shown evidences that recombination, mutation, and gene density are important determinants of the rate of adaptive evolution within the *Drosophila* genome.

## Materials and Methods

### Population Genomic Data, Polymorphism, and Divergence Estimates

This study was carried out on the four large autosomes (2L, 2R, 3L, and 3R) of *D. melanogaster* using release 5 of the Berkeley *Drosophila* Genome Project (BDGP 5, http://www.fruitfly.org/sequence/release5genomic.shtml, last accessed May 2010) as the reference genome.

### North American Population

The population genomic data comes from Raleigh, North Carolina. The details of their provenance and breeding are in Mackay et al. (2012), Freeze 1.0 DGRP project. Sites with residual heterozygosity and low quality values were excluded from the analyses. The method for jointly estimating the DFEs on new mutations and the rate of adaptive substitution requires all sites to have been sampled in the same number of chromosomes (DFE-alpha method, Eyre-Walker and Keightley 2009; see below) and since some sites were not successfully sampled in all samples, we reduced the original data set to 128 isogenic lines by randomly sampling the polymorphisms at each site without replacement. To estimate divergence out to *D. yakuba* we sampled randomly one *D. melanogaster* single chromosome.

Coding exon and short intron (≤65 bp) annotations from *D. melanogaster* were retrieved from FlyBase (release 5.50, http://flybase.org/, last accessed March 2013). Genes 1:1 orthologs across *D. yakuba*–*D. melanogaster* were obtained from FlyBase (http://flybase.org/). We used *D. yakuba* as the outgroup species since there is less chance of ancestral polymorphism contributing to divergence, avoiding in this way the effect of low divergence affecting the estimates of adaptive evolution (Keightley and Eyre-Walker 2012). We obtained a multiple genome alignment between the DGRP isogenic lines (Mackay et al. 2012) and the *D. yakuba* genome (Clark et al. 2007) using the BDGP 5 coordinates. This alignment is publicly available at http://popdrowser.uab.cat/ (last accessed May 2010) (Ràmia et al. 2012)*.* For each gene we took all nonoverlapping coding exons, independently of their inclusion levels. When two exons overlapped, the largest was chosen for subsequent analyses. Only exons without frameshifts, gaps or early stop codons were retained. In this way, we tried to avoid potential alignment errors will inflate our mutation and adaptation rate estimates and create an artifactual positive correlation between them. Our final data set fulfilling all these criteria had 6,141 coding genes.

Exonic sequences were trimmed in order to contain only full codons. We defined our sites “physically,” so we estimated the rates of substitution at sites of different degeneracy separately. Only 0-fold and 4-fold degenerate sites in exon core codons (as described by Warnecke and Hurst 2007) were used. To estimate the rate of synonymous substitutions, we restricted our analysis to those triplets coding the same amino acid in the two species (*D. melanogaster*–*D. yakuba*). In restricting our analysis to codons not exhibiting nonsynonymous differences we assume that the codon has undergone no amino acid substitution—this avoids having to compute the different pathways between two codons, which differ by more than one change and it is a reasonable assumption given the low level of amino acid divergence. For 4-fold degenerate sites we used the method of Tamura (1992) to correct for multiple hits; this method allows for unequal GC content and ts/tv bias. Jukes and Cantor substitution method was used to correct for multiple hits at 0-fold sites (Jukes and Cantor 1969). We calculated the number of substitutions and the folded SFS for 4-fold degenerate sites and 0-fold degenerate sites, using an ad hoc Perl Script.

Following Halligan and Keightley (2006), in this study we used positions 8–30 of introns ≤65 bp in length as an alternative neutral reference for some analyses. For intron sequences, the invariant GT and AG dinucleotides at the 5′- and 3′-splice junctions, respectively, were excluded before calculating divergence. Only genes with at least two short introns and with less than 10% of gaps in the aligned sequences were kept. 3,369 orthologous genes passed the intron quality criteria in our final data set. We used an ad hoc Perl Script to estimate the number of short intron substitutions and to compute the folded SFS. Multiple hits were corrected using Jukes and Cantor method (Jukes and Cantor 1969).

### African Population

We also used population genomic data from an African population. This comes from Gikongoro, Rwanda (DPGP2, Pool et al. 2012). The details of the assembly and data filtering can be found in Campos et al. (2014). The number of synonymous and nonsynonymous sites and substitutions (computed by the Comeron 1995 method, which defines a site as a “mutational opportunity”) and the SFS for 7,231 autosomal coding genes were estimated by Campos et al. (2014) and details are provided there. We study only those genes shared by both data sets (DGRP and DPGP2) taking into account the differences in gene annotation versions. This resulted in a data set of 4,283 autosomal genes coming from this data set.

### Codon Bias Estimates, Recombination Landscape, and Gene Density Estimates

We used the CodonW software (http://codonw.sourceforge.net/ [last accessed June 2012] by Peden [1999]) to estimate the *Fop.* A higher *Fop* value suggest a higher efficacy of selection for codon usage, and vice versa. Recombination rates were taken from Comeron et al. (2012) (www.recobinome.com). They estimated the rate of crossovers in 100 kb nonoverlapping windows in cM/Mb units. The rate of crossing-over for a gene was the rate in the 100 kb that overlapped the midpoint of the gene. Unlike Campos et al. (2014), we did not apply LOESS regression to smooth out the recombination landscape, as we were interested in the fine-scale effects of recombination on the *D. melanogaster* genome. We use all the coding genes from our annotation file (release 5.50) to estimate gene density. Hence, our estimates of gene density are not based only on the 6,141 genes present in our data set. To compute gene density we first calculate the midpoint coordinate of each gene; the start point corresponds to the first position of the first coding exon and the stop point corresponds to the last position of the last coding exon. Then we count all coding sites 50 kb upstream and 50 kb downstream the midpoint coordinate and we use this coding sequences count as an estimate of gene density. Thus, each gene has its own gene density estimate.

### Testes, Immune Genes, and Permutation Test

If immune and male-biased or testes specific genes tend to be overrepresented in specific recombination, mutation, or gene density regions then the correlations to the rate of adaptive evolution would not necessarily be a consequence of adaptive evolution being affected by recombination, mutation, or gene density. Thus, Gene Ontology (GO) terms for 6,141 genes were downloaded from Fruitfly release 78 using the R package biomaRt (Durinck et al. 2005). A list of GO terms related to immune response and testes was constructed using the EBI’s GO tool QuickGO (Binns et al. 2009). When a given gene was associated to a GO term from this list it was labeled as “Immune&Testes genes,” the rest of genes were labelled as “Control genes.” The list of immune response and testes related GO terms and the lists of genes in each group can be consulted in the supplementary table S6, Supplementary Material online. A permutation test was applied to assess whether *K_a_*_+_ are significantly higher as it has been reported before (Pröschel et al. 2006; Haerty et al. 2007; Obbard et al. 2009) for immune response and testes related genes relative to the rest of control genes. We shuffled without replacement 1,000 times the complete list of genes by means of ad hoc Bash and Perl Scripting. Then, we estimated *K_a_*_+_ using the DFE-alpha software (Eyre-Walker and Keightley 2009, see below) for each randomized group. Thus, we got the expected null distribution for the differences between Control genes minus the Immune&Testes genes for the statistic *K_a_*_+_. Finally, the one-tailed *P* value was obtained by counting the number of replicates below the observed difference divided by the total number of replicates (1,000). The expected null distributions and the observed differences can be consulted in the supplementary figure S4, Supplementary Material online.

### Gene Bins and Adaptation Estimates

To estimate the rate of adaptive evolution it is necessary to combine data from several genes because estimates from a single gene are noisy and often undefined because of the lack of segregating (or divergent) sites for some site classes. We therefore grouped genes into bins according to their rate of recombination, mutation rate, gene density, and/or *Fop.* The rank of values for all these bins can be consulted in the supplementary material, Supplementary Material online.

It is essential to have a selection-free reference sequence that can be used as a baseline for determining the rate of adaptive substitution acting on a particular target sequence (in our case 0-fold degenerate sites). In this study, we used the exon core 4-fold degenerate sites as the main proxy for the neutral mutation rate. For some cross-validation analyses short intron sites were also used. DFE-alpha (Eyre-Walker and Keightley 2009) models the DFE at functional sites by a gamma distribution, specified by the mean strength of selection, γ = −Nes, and a shape parameter β, allowing the distribution to take on a variety of shapes ranging from leptokurtic to platykurtic. DFE-alpha can model a single, instantaneous change in population size from an ancestral size *N*_1_ to a present-day size *N*_2_ having occurred *t*_2_ generations ago. Provided the SFS at both neutral and functional sites and the respective levels of divergence, DFE-alpha infers γ, β, *N*2/*N*1, *t*_2_, and α at functional sites. From these estimates *K_a_*_+_ can be easily estimated with the expression: *K_a_*_+_ = α × *K_a_.* We ran DFE-alpha for each bin independently using the local version provided at: http://www.homepages.ed.ac.uk/pkeightl//software. DFE-alpha was run in the folded SFS mode as the results are more robust.

### Hypergeometric Sampling

To analyze the role of mutation on adaptation we correlated 4-fold divergence (*K*_4_) to the rate of adaptive 0-fold substitutions (*K_a_*_+_). A limitation here is that *K*_4_ and *K_a_*_+_ are not independent, since the estimation of *K_a_*_+_ depends on *K*_4_, and then we expect *K_a_*_+_ and *K*_4_ to be negatively correlated just through sampling error. To overcome this problem we split our mutation rate estimate (*K*_4_) into three independent variables (similar to the splitting done in Smith and Eyre-Walker 2002; Piganeau and Eyre-Walker 2009; Stoletzki and Eyre-Walker 2011; Gossmann et al. 2012). This was done by generating a random multivariate hypergeometric variable as follows:

(1) $$D_{4,1}=\text{multivariateHypergeometric}(D_{4},0.33\times L_{4}),$$

(2) $$D_{4,2-3}=D_{4}-D_{4,1},$$

(3) $$D_{4,2}=\text{ multivariateHypergeometric}(D_{4,2-3},\;0.33\times L_{4}),$$

(4) $$D_{4,3}=D_{4}-D_{4,1}-D_{4,2}$$

where *L_4_* is the number of 4-fold sites and **D**_4_ is the total number of 4-fold divergent sites. We divided **D**_4,1_, **D**_4,2_ and **D**_4,3_ by ⅓ × *L_4_* to get *K*_4,1_, *K*_4,2,_ and *K*_4,3_, respectively. We used *K*_4,1_ to rank genes and assign genes to bins, we then used *K*_4,2_ to estimate the rate of adaptive nonsynonymous substitution (*K_a_*_+_) and *K*_4,3_ as an estimate of the mutation rate.

To test if genes with high mutation rates have lost more adaptive amino acid substitutions than genes with low mutations rates due to HRi, we have categorized genes into low and high mutations groups after splitting K_4_ into two statistically independent variables; *K*_4,1_ was used to rank genes and assign genes to bins, and *K*_4,2_ was used to estimate *K_a_*_+_. Again, this was done by generating a random multivariate hypergeometric variable as follows:

(5) $$D_{4,1}=\text{ multivariateHypergeometric}(D_{4},\;0.5\times L_{4}),$$

(6) $$D_{4,2}=D_{4}-D_{4,1},$$

### Estimating the Number of Substitutions Lost to HRi

To estimate how many adaptive substitutions are lost to HRi, we proceeded as follows. Let *K_a+(i)_*, *L_a(i__)_*_,_ and *RR_(i_*_)_ be the estimated rate of *K_a__+_*, the total number of 0-fold sites and the average rate of recombination for the *i*th group of genes (grouped by recombination rate). We fit a LOESS curve to the relationship between *K_a+_* and the rate of recombination. Let the estimated value of *K_a+_* for the *i*th group of genes from the LOESS curve be *K_a__+(i)′_*—this can be thought of as the predicted mean rate of adaptive nonsynonymous substitution for genes of the observed recombination rate. We took the average *K_a+_* for genes with rates of recombination above 2 cM/MB as our estimate of the rate of adaptive nonsynonymous substitution without HRi—let this be *K_a+,_*_no_HRi_. The expected total number of adaptive nonsynonymous substitutions without any HRi is therefore:

(7) $${\text{Total}}_{K_{a+,{\text{no}}_{\text{HRi}}}}=\sum^{\;}\left(L_{a\left(i\right)}\times K_{a+,{\text{no}}_{\text{HRi}}}\right),$$

and the number lost adaptive substitutions to HRi is:

(8) $$\text{Total}\_K_{a+,\text{ lost}}=\sum^{\;}\left(L_{a\left(i\right)}\times \left(K_{a+,\text{ no}\_\text{HRi}}-K_{a+,{\left(i\right)}^{\prime }}\right)\right),$$

for groups of genes with a rate of recombination less than 2 cM/MB. Because the mean rate of adaptive nonsynonymous substitution from the LOESS curve can be negative we repeated the analysis setting any value of *K_a+(i)′_* to zero if it was less than zero. Finally the proportion of substitutions lost to HRi:

(9) $$f_{\text{HRi}}=\frac{\text{Total}\_K_{a+,\text{ lost}}}{\text{Total}\_K_{a+,\text{ no}\_\text{HRi}}}$$

### Confidence Intervals, Bootstraps, and P values

To calculate the 95% CIs for the proportion of adaptive amino acid substitutions lost due to interference (*f*_HRi_), we bootstrapped 1,000 times the data by gene. We split each 1,000 random data sets into 45 recombination bins (containing 136 genes each) and reestimated *K_a_*_+_ for each bin independently using the DFE-alpha software (Eyre-Walker and Keightley 2009, see above). For each random data set, we fitted a LOESS curve to the relationship between *K_a_*_+_ and the rate of recombination and reestimated the proportion of substitutions lost to HRi, *f*_HRi_ (see above).

For testing if genes undergoing high mutation rates (and/or high gene density) have lost more adaptive substitutions than genes under low mutation rates (and/or under low gene density), we took the data set bootstrapped above and for each bootstrap replicate, we split the data set first by its gene density and then by its *K*_4_. Before splitting by *K*_4_ we split gene's *K*_4_ estimates into two variables; *K*_4,1_ and *K*_4,2_ sampling from an hypergeometric distribution (see the details of this sampling above). Being more specific, for each bootstrap replicate we: 1) took the 50% of the genes with the highest (and lowest) gene density, 2) within each gene density group we took the 50% of the genes with the highest (and lowest) *K*_4,1_ to define the high (and low) mutation group, 3) we divided each mutation group into 12 recombination rate bins (of 128 genes each), and 4) we estimated *K_a_*_+_ using *K*_4,2_ for each recombination—mutation—gene density group. The distribution of *f*_HRi_ for each gene category was obtained by applying expression (9) to each bootstrap replicate. Thus we have 1,000 *f*_HRi_ estimates for each gene category: GenH-MutH (high gene density and high mutation rate genes), GenH-MutL (high gene density and low mutation rate genes), GenL-MutH (low gene density and high mutation rate genes), and GenL-MutL (low gene density and low mutation rate genes). To test if *f*_HRi_ differs significantly across gene categories we estimated the statistic *Z* with the following expression:

(10) $$Z=f_{\text{HRi }\left(\text{GenH}-\text{MutH}\right)}-f_{\text{HRi }(}{}_{\text{GenL}-\text{MutL})},$$

where *f*_HRi (GenH-MutH)_ is the proportion of lost substitutions for genes with high gene density and high mutation rates and *f*_HRi (GenL-MutL_*_)_* is the proportion of lost substitutions for genes with low gene density and low mutation rates. We did all the combinations among the four gene categories to obtain six different Z distributions. Finally, for each Z distribution (or comparison between gene categories) the one-tailed *P* value was estimated as the proportion of the *Z* distribution below (or above) zero. Likewise, to test whether the average *K_a_*_+_ differed between gene categories we substitute the *f*_HRi_ by the average *K_a_*_+_ in expression (10).

### Statistical Analyses

All statistical analyses were performed using the R statistical package (R Core Team 2013). ANCOVAs and multiple linear regressions were carried out calling the R function “lm” (from the R package “base”). Linear and nonlinear regression were run using the R function “nls” (from the R package “stats”). To compare the linear and curvilinear model fit we used the R function “ANOVA” (from the R package base). We calculated Spearman's rank correlations (*ρ*_s_) using the basic R function “cor.test” (from the R package base). The random hypergeometric variable was obtained through the R function “rhyper” (from the R package stats). LOESS regression was run using the R package stats after setting the smoothness parameter “span” from the default 0.75 value to 1. Increasing the span parameter decreases the smoothness of the fitted curve making the regressions more robust (or less noisy) across bootstrap replicates. All these R scripts are available upon request.

## Supplementary Material

Supplementary figures S1–S8 and tables S1–S6 are available at *Molecular Biology and Evolution* online (http://www.mbe.oxfordjournals.org/).

Supplementary Data
