# Supplementary material for: Adaptive Evolution Is Substantially Impeded by Hill–Robertson Interference in Drosophila
Source: Mol Biol Evol. 2015 Oct 22;33(2):442–55. doi: 10.1093/molbev/msv236 (PMC4794616; doi:10.1093/molbev/msv236)
Supplement: Supplementary Data [file supp_33_2_442__index.html]

Adaptive Evolution Is Substantially Impeded by Hill–Robertson Interference in Drosophila — Adaptive Evolution Is Substantially Impeded by Hill–Robertson Interference in Drosophila — Supplementary Data 

# Adaptive Evolution Is Substantially Impeded by Hill–Robertson Interference in *Drosophila*

## Supplementary Data

files

- Supplementary Data - pdf file
- Supplementary Data - xls file
