# Supplementary material for: Adaptive Evolution Is Substantially Impeded by Hill–Robertson Interference in Drosophila
Source: Mol Biol Evol. 2015 Oct 22;33(2):442–55. doi: 10.1093/molbev/msv236 (PMC4794616; doi:10.1093/molbev/msv236)
Supplement: Supplementary Data [file supp_msv236_Supplementary_Figures.pdf]

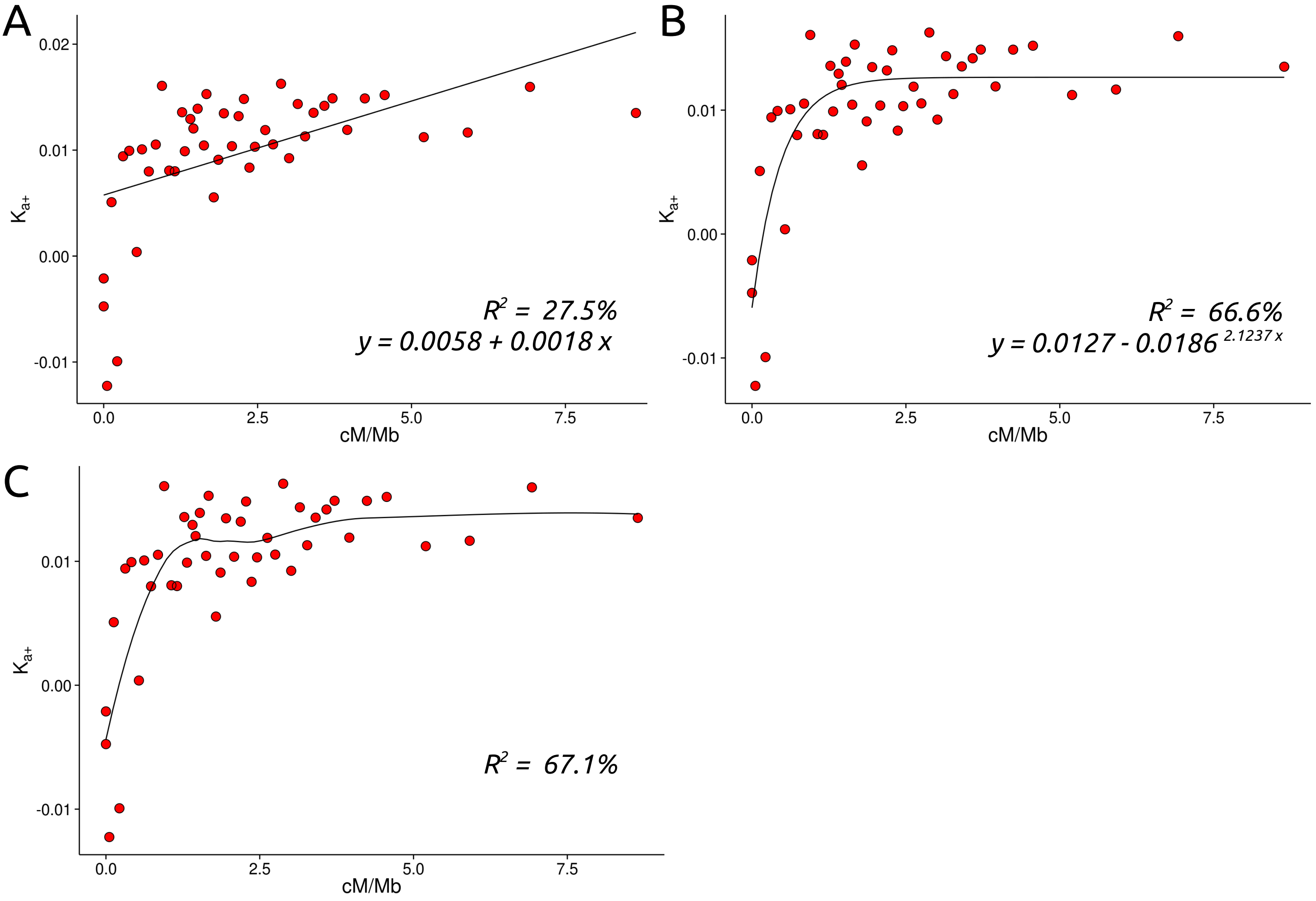

Supplementary Fig. 1. Relations between K_{a+} in the *y* axis and the rate of recombination (cM/Mb) in the *x* axis: (A) fitting a linear regression, (B) fitting a non-linear regression and (C) fitting a local regression (LOESS). Each data point has been estimated binning 136 genes according to their recombination rate levels. The average recombination rate and K_{a+} estimate for each bin can be consulted in the supplementary table 1. R^2: proportion of explained variance.

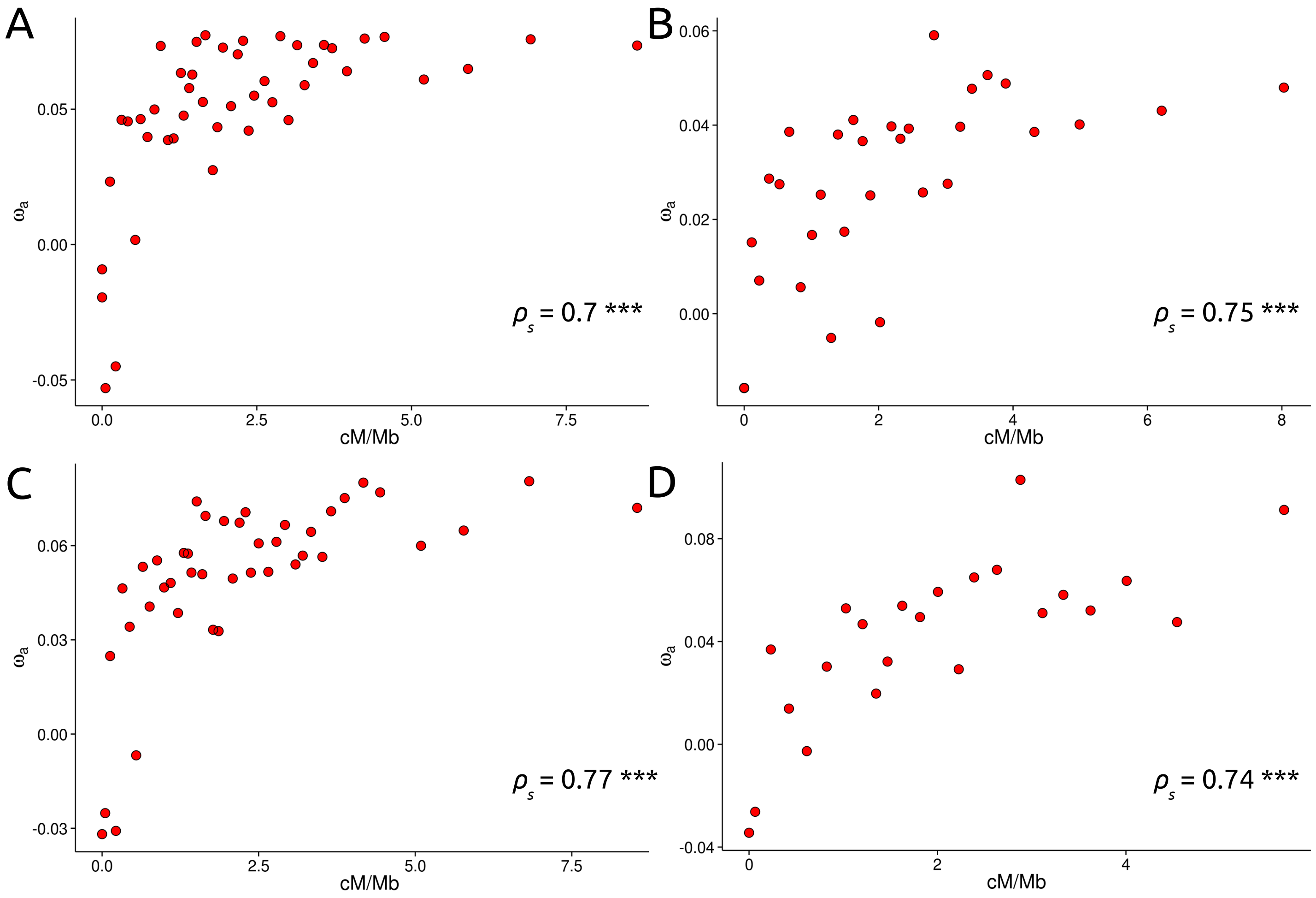

Supplementary Fig. 2. Relations between ω_A in the *y* axis and the rate of recombination (cM/Mb) in the *x* axis: (A) using DGRP data, North Carolina population, (B) using DPGP2 data, Rwanda population, (C) using DGRP data excluding immune response and testes related genes and (D) using DGRP data and short intron sites as neutral reference (< 66 nt, bases from 8 to 30). Each data point has been estimated binning 136 genes according to their recombination rate levels. The average recombination rate and ω_A estimate for each bin can be consulted in the supplementary table 1. ρ_s: Spearman's rank correlation coefficient, with significance denoted by asterisks (***<0.001; **<0.01; *<0.05).

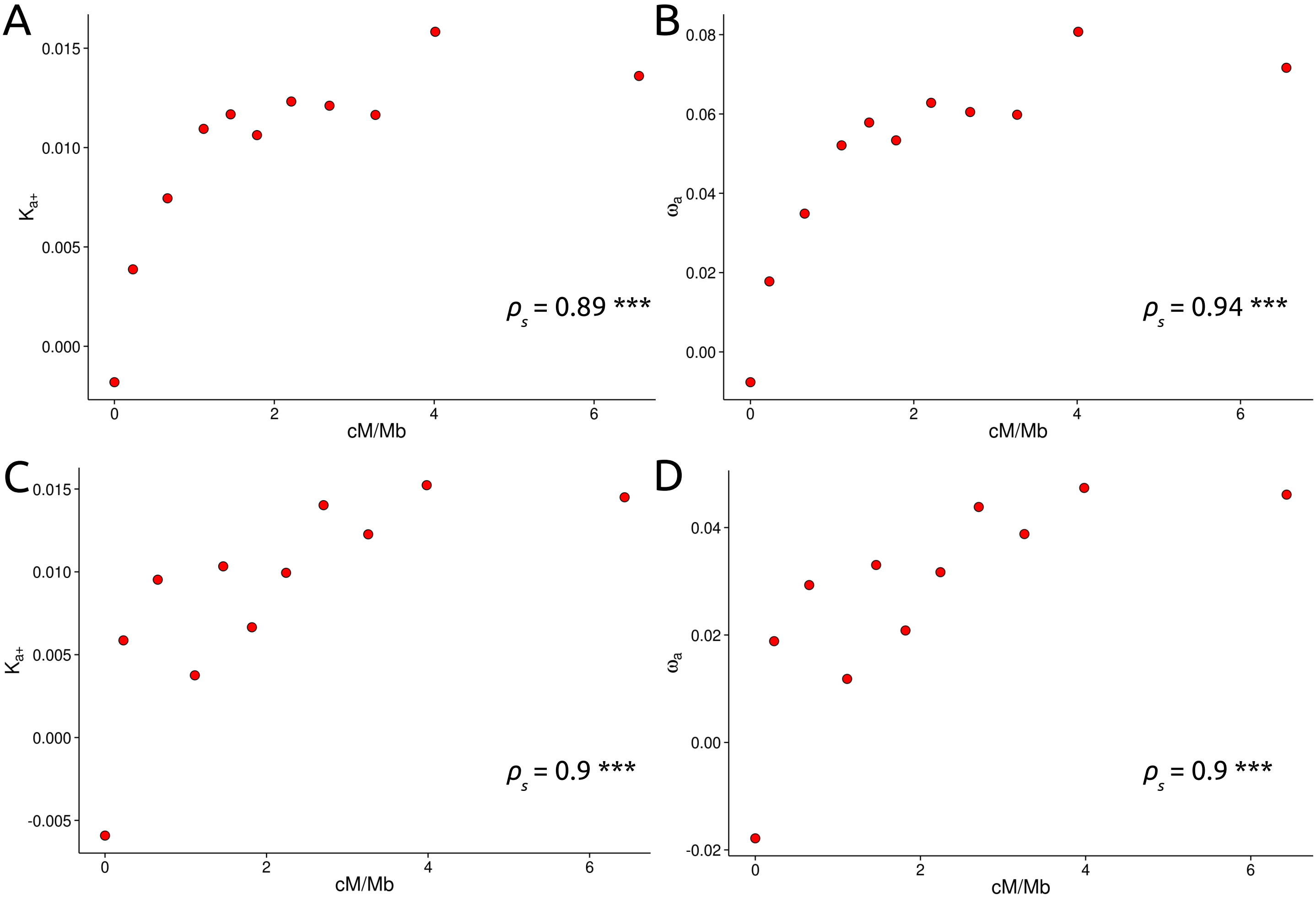

Supplementary Fig. 3. Relationship between K_{a+} (A and C) or ω_A (B and D) in the *y* axis and the rate of recombination (cM/Mb) in the *x* axis binning genes as in Campos et al. (2014) using: (A-B) using DGRP data, North Carolina population and (C-D) using DPGP2 data, Rwanda population. The average recombination rate and K_{a+} (and ω_A) estimate for each bin can be consulted in the supplementary table 2. Table 1 shows the R^2, AIC and inferred parameters for the linear and nonlinear regression equations. ρ_s: Spearman's rank correlation coefficient, with significance denoted by asterisks (***<0.001; **<0.01; *<0.05).

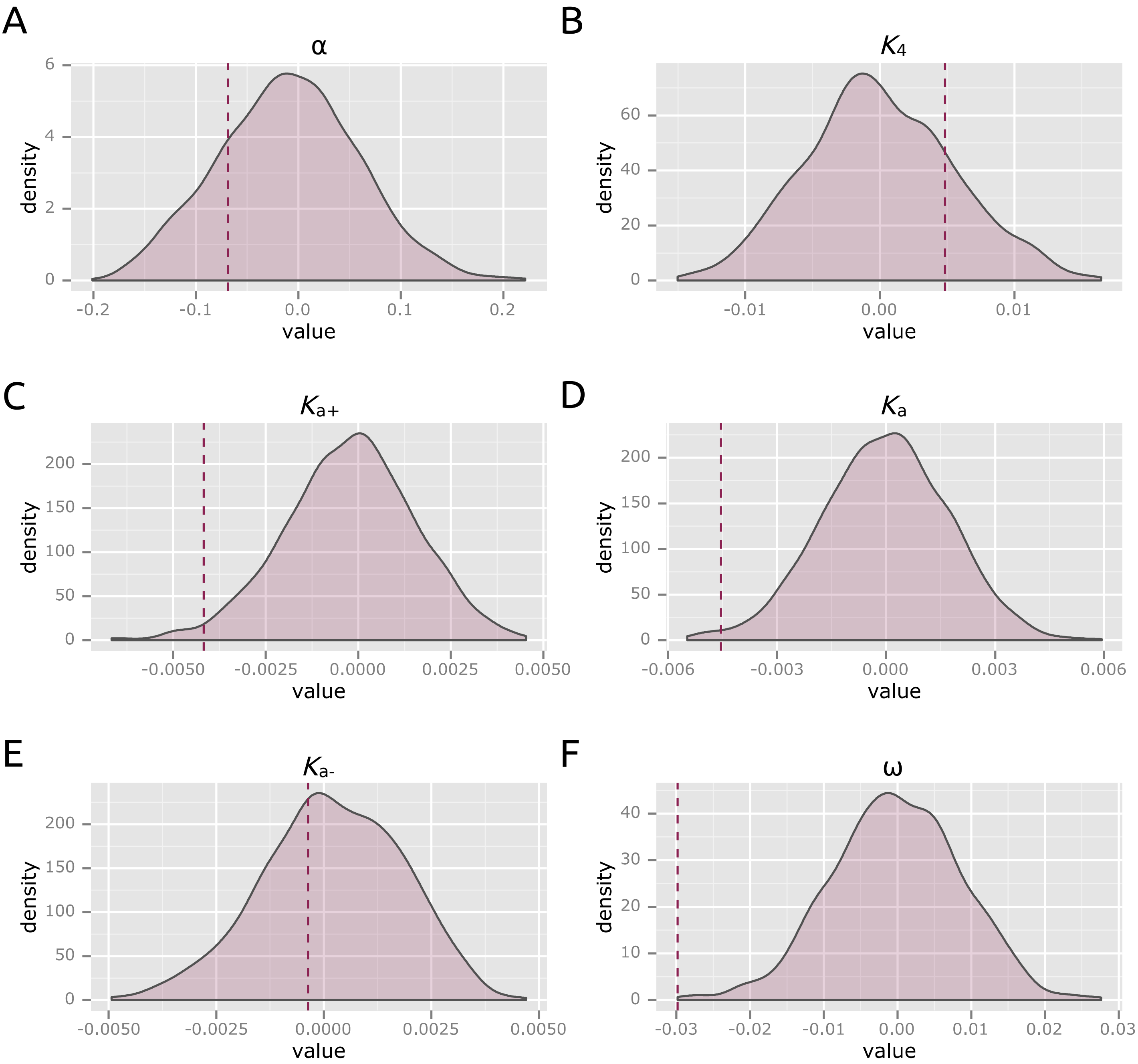

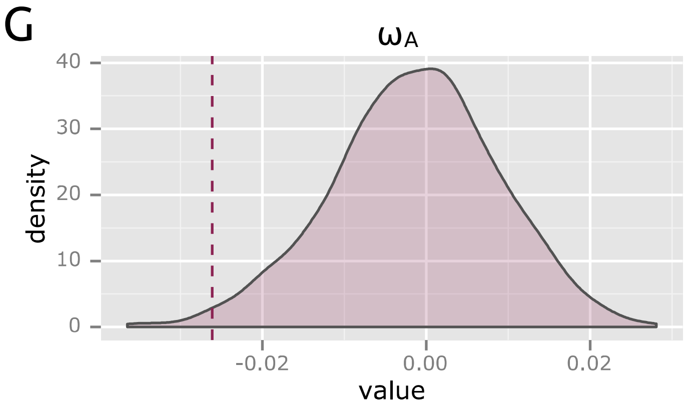

H

Statistic	p-value
α	0.198
K_{a+}	0.017
K_{a-}	0.360
ω_a	0.010
K_4	0.187
K_a	0.009
ω	0.001

Supplementary Fig. 4. Permutation test null distributions and observed differences (red dashed vertical lines) between “Control genes” and “Immune&Testes genes” based on several statistics: (A) α, the proportion of adaptive nonsynonymous substitutions, (B) K_4, the number of 4-fold synonymous substitutions per site, (C) K_{a+}, the number of adaptive non-synonymous substitutions per site, (D) K_a, the number of non-synonymous substitutions per site, (E) K_{a-}, the number of non-adaptive non-synonymous substitutions per site, (F) ω (K_a/K_4), (G) ω_A (K_{a+}/K_4) and (H) the one-tailed p-value for the different statistics.

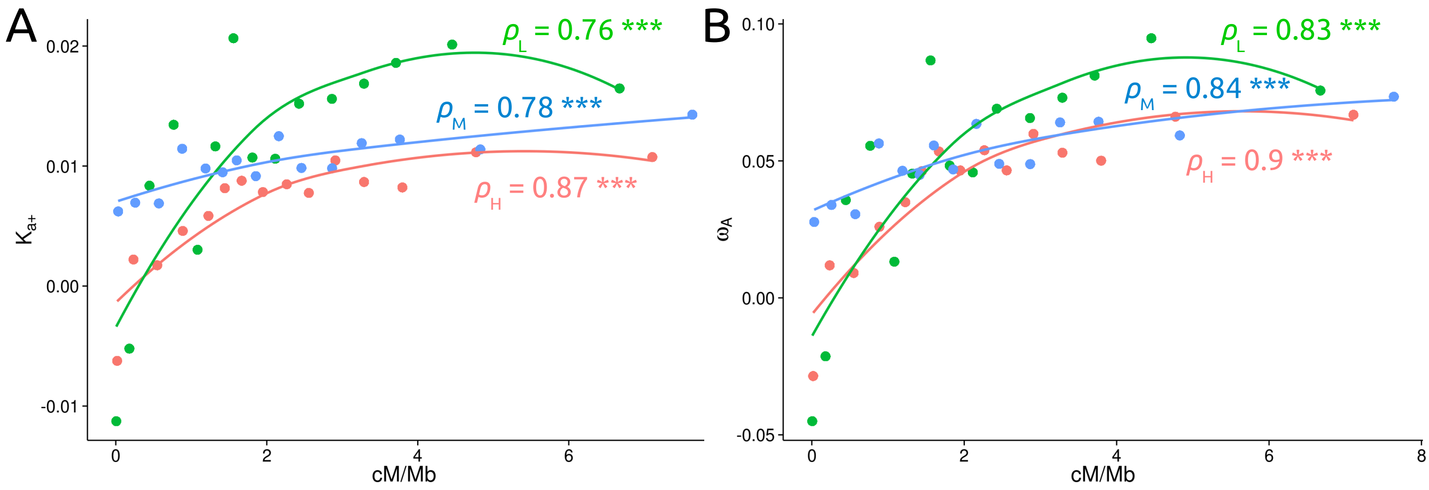

Supplementary Fig. 5. Relationship between (A) K_{a+} and (B) ω_A in the *y* axis and the rate of recombination (cM/Mb) in the *x* axis for three *Fop* groups, respectively. Genes belonging to the high *Fop* (H) group are in red, medium *Fop* (M) genes are in blue and low *Fop* (L) genes are in green. Each data point has been estimated binning 136 genes according to their recombination rate and *Fop* levels. The average recombination rate, *Fop* and K_{a+} (and ω_A) estimate for each bin can be consulted in the supplementary table 3. Table 1 shows the R^2, AIC and inferred parameters for the linear and nonlinear regression equations for each *Fop* group. ρ_s: Spearman's rank correlation coefficient, with significance denoted by asterisks (***<0.001; **<0.01; *<0.05). The lines are LOESS regressions.

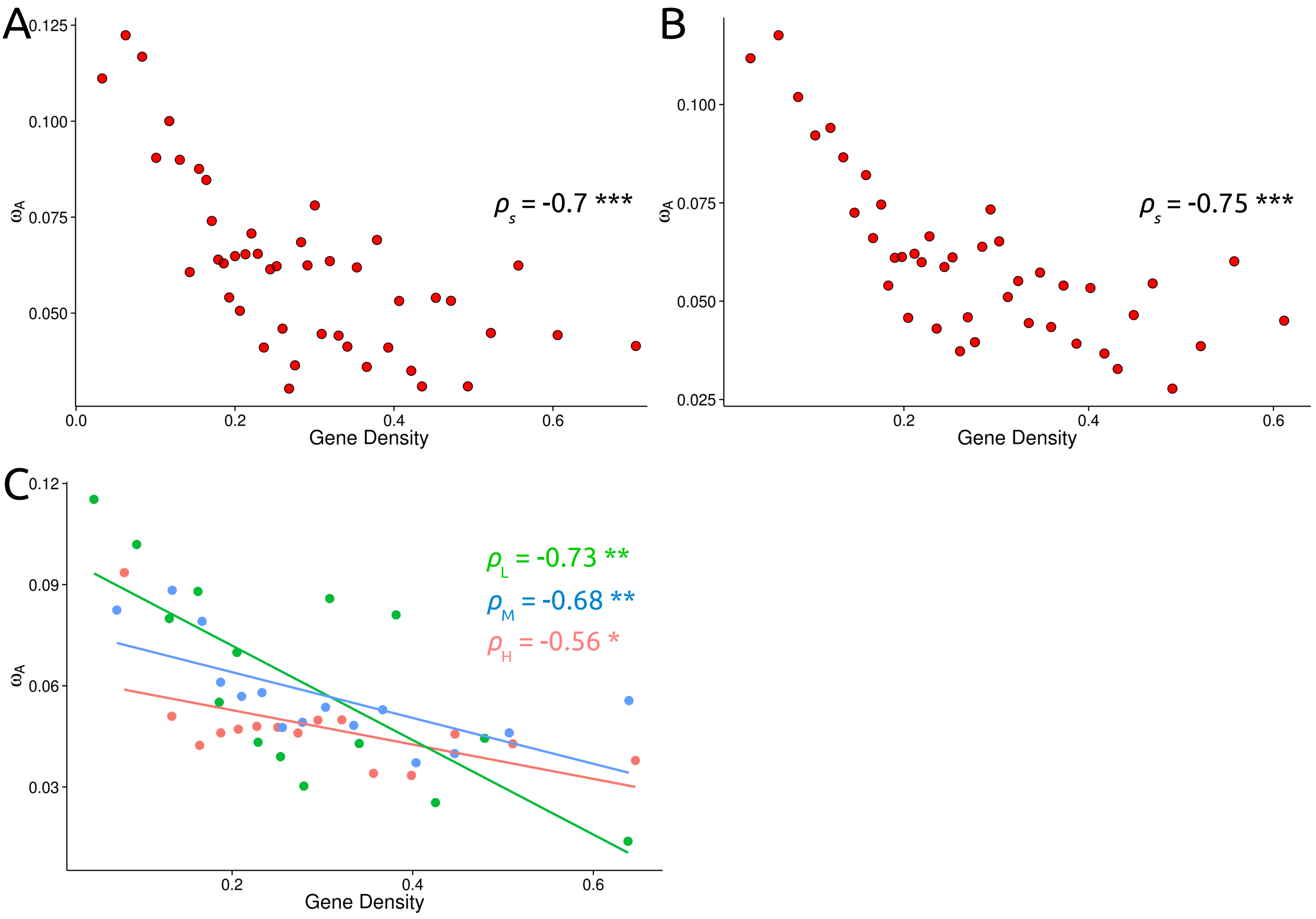

Supplementary Fig. 6. Relationship between ω_A in the y axis and the gene density in the x axis: (A) using DGRP data, North Carolina population, (B) excluding immune response and testes related genes and (C) splitting the dataset into three *Fop* groups. Genes belonging to the high (H) group are in red, medium (M) genes are in blue and low (L) genes are in green. Each data point has been estimated binning 136 genes according to their gene density and/or *Fop*. The average gene density, *Fop* and estimated ω_A for each bin can be consulted in the supplementary table 4. ρ_s: Spearman's rank correlation coefficient, with significance denoted by asterisks (***<0.001; **<0.01; *<0.05; . 0.1-0.05). The lines are least-squares regressions but should be regarded only as indicative, in view of the binning of the data.

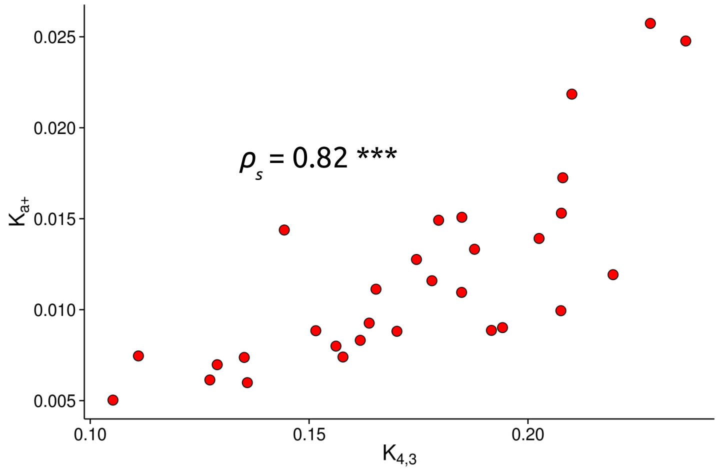

Supplementary Fig. 7. Relationship between K_{a+} in the *y* axis and an estimate of the mutation rate ($K_{4,3}$) in the *x* axis after excluding genes with low recombination rates (< 1.32 cM/Mb). Each data point has been estimated binning 136 genes according to their mutation rate ($K_{4,1}$). The average mutation rate ($K_{4,3}$) and K_{a+} estimate for each bin can be consulted in the supplementary table 5. ρ_s: Spearman's rank correlation coefficient, with significance denoted by asterisks (***<0.001; **<0.01; *<0.05).

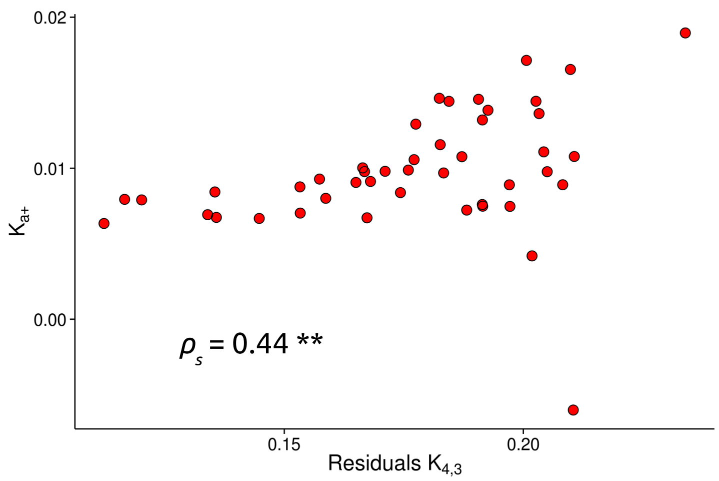

Supplementary Fig. 8. Relationship between K_{a+} in the *y* axis and the residuals of the multiple linear regression: $K_{4,3}$ ~ Recombination Rate + *Fop*, in the *x* axis. The residuals are an alternative estimate of the mutation rate which controls for differences in the rate of recombination and *Fop*. Each data point has been estimated binning 136 genes according to their mutation rate ($K_{4,1}$). The average mutation rate (residuals $K_{4,3}$) and K_{a+} estimate for each bin can be consulted in the supplementary table 5. ρ_s: Spearman's rank correlation coefficient, with significance denoted by asterisks (***<0.001; **<0.01; *<0.05).
